# A General Latent Class Model for Performance Evaluation of Diagnostic Tests in the Absence of a Gold Standard: An Application to Chagas Disease

**DOI:** 10.1155/2012/487502

**Published:** 2012-07-31

**Authors:** Gilberto de Araujo Pereira, Francisco Louzada, Valdirene de Fátima Barbosa, Márcia Maria Ferreira-Silva, Helio Moraes-Souza

**Affiliations:** ^1^Department of Education and Nursing Community Health, Federal University of Triângulo Mineiro, 38025-180 Uberaba, MG, Brazil; ^2^Department of Applied Mathematics and Statistics, ICMC, University of São Paulo, 13560-970 São Carlos, SP, Brazil; ^3^Research Group for Blood Transfusion Security, Federal University of Triângulo Mineiro, 38025-180 Uberaba, MG, Brazil; ^4^Postgraduate Student in Clinical Pathology, Federal University of Triângulo Mineiro, 38025-180 Uberaba, MG, Brazil; ^5^Discipline of Hematology, Federal University of Triângulo Mineiro, 38025-180 Uberaba, MG, Brazil

## Abstract

We propose a new general Bayesian latent class model for evaluation of the performance of multiple diagnostic tests in situations in which no gold standard test exists based on a computationally intensive approach. The modeling represents an interesting and suitable alternative to models with complex structures that involve the general case of several conditionally independent diagnostic tests, covariates, and strata with different disease prevalences. The technique of stratifying the population according to different disease prevalence rates does not add further marked complexity to the modeling, but it makes the model more flexible and interpretable. To illustrate the general model proposed, we evaluate the performance of six diagnostic screening tests for Chagas disease considering some epidemiological variables. Serology at the time of donation (negative, positive, inconclusive) was considered as a factor of stratification in the model. The general model with stratification of the population performed better in comparison with its concurrents without stratification. The group formed by the testing laboratory Biomanguinhos FIOCRUZ-kit (c-ELISA and rec-ELISA) is the best option in the confirmation process by presenting false-negative rate of 0.0002% from the serial scheme. We are 100% sure that the donor is healthy when these two tests have negative results and he is chagasic when they have positive results.

## 1. Introduction

 A major challenge for diagnostic medicine is the determination of the true health status of an individual (sick or healthy) in relation to a certain disease when the gold standard test does not exist or its use is limited. Such problem may occur especially when the gold standard technique is an invasive procedure, risky, or financially costly and the definitive verification for apparently healthy individuals is thus neither practical nor ethical.

The development of statistical procedures for the estimation of performance parameters (sensitivity and specificity) of diagnostic tests is relatively straightforward when the gold standard exists and is applied in all subject of sample under investigation (see [1–3]). When the gold standard test does not exist or its use is limited, the most widely used alternative is the latent class modeling [4–8].

The latent class structure allows for modeling flexibility for both frequentist and Bayesian approaches, and the estimates of interest can be obtained based on numerical methods. For instance, on has expectation-maximization algorithms [9] in the case of the frequentist approach and Gibbs sampling [10] and Metropolis-Hastings [11] algorithms in the case of the Bayesian approach.

Considering the conditional independence structure between tests, that is, given the health condition of the subject, the result of test *k* is independent of the result of test *k*′, several researchers have developed and implemented models that evaluate the performance of one or multiple diagnostic tests considering a model structure with partial or complete absence of a gold standard, sample stratification, and inclusion of covariates.

Several of these studies have tested models in which only subjects with positive results in at least one of the tests are submitted to the gold standard. For instance, Schatzkin et al. introduced a model to evaluate the relative performance of two tests using a frequentist approach [12]. In a simulation study, Cheng et al. evaluated the average coverage probability of the confidence intervals of these model parameters [13], while Pepe and Alonzo proposed the inclusion of a vector of covariates in this model [14]. Also, using a frequentist approach, Macaskill et al. presented a model for the estimation of point and interval likelihood ratios [15] and Walter proposed an alternative model able to directly estimate these measures from the latent class [6]. The Bayesian extensions of this model has developed by Van der Merwe and Maritiz [16] and Martinez et al. [17].

Situations in which none of the sample subjects are submitted to the gold standard are also widely reported in the literature. Gastwirth et al. proposed Bayesian modeling for the case of only one test and low disease prevalence, and they called attention to the fact that care should be taken when choosing a prior distribution [18]. Johnson and Gastwirth added transformations in the parameters to this model [19]. Joseph et al. applied Bayesian modeling based on the Gibbs sampling algorithm to the case of two diagnostic tests [4]. Leisenring and Pepe developed a logistic regression model for the case of one test in the presence of a covariate vector [20]. Using a frequentist approach, Albert et al. proposed a model including a finite mixture of distributions and covariates based on a random effect model [21]. Rathouz et al. presented a conditional logistic regression model including missing covariate data [22]. Martinez et al. first developed an extension of the model proposed by [4] including a covariate vector based on Metropolis-Hasting algorithms [23], and then a generalization to the case of *L* tests and *M* covariates, which were applied to a problem with three diagnostic tests (*L* = 3) and two covariates (*M* = 2) [8].

To evaluate the performance of two conditionally independent diagnostic tests, Hui and Walter developed, from the frequentist point of view, a model based on the stratification of the sample into two strata, considering different disease prevalences in each stratum but similar sensitivity and specificity values [24]. Johnson and Gastwirth discussed the applicability of this method to other datasets [25]. Enøe et al. proposed a Bayesian extension of this model based on a Gibbs sampling scheme [7]. According to Toft et al., it is important to find a stratifying factor which does not violate the assumption of constant sensitivity and specificity among the strata. When this assumption is questionable is indicated find another stratifier factor, preferably from a practical criterion, and to consider a model that allows inclusion of covariates [26].

The model in [24] is known as the Hui-Walter paradigm and has been widely discussed and applied in the literature using frequentist and Bayesian approaches, (see e.g., [8, 17, 23, 26–34], among others), but without including the general case of *L* tests, *M* covariates, and *V* strata in the model structure which is properly proposed here.

Based on the above statement, the main aim of this paper is to present a Bayesian latent class approach when any of the subjects of the sample under investigation is subjected to the gold standard for evaluating the performance of *L* imperfect tests applied to *V* subsets and considering the presence of *M* covariates.

The paper structure is as follows. Our general model is present in Section 2, including the covariate case as well as the inferential procedure.  Section 3 presents the results of a small simulation study where the general model is compared to its concurrent particular cases.  Section 4 presents the results of our modeling applied to a real dataset on Chagas' disease.  Section 5 presents the final comments.

## 2. Model: Full Absence of Gold Standard

We present in this section a latent class modeling considering the population stratified on the assumption of Hui and Walter [24] and full absence of gold standard.

### 2.1. Performance Parameters

In the study of the performance of diagnostic tests, the probability of a test *k* to yield a positive result (*T*_*k*_ = 1), given that the individual has the disease (*D* = 1), is known as the sensitivity of test and is mathematically expressed as Se_*k*_ = *P*(*T*_*k*_ = 1 | *D* = 1), *k* = 1,2,…, *L*. If test *k* is not 100% sensitive, it will fail to detect the disease in some individuals known to be sick. The proportion of negative results of test among sick individuals is known as the false-negative rate (FNR_*k*_ = 1 − Se_*k*_).

The specificity of test *k* refers to the probability of this test to yield a negative result (*T*_*k*_ = 0), given that the individual does not have the disease (*D* = 0), and it is expressed as Sp_*k*_ = *P*(*T*_*k*_ = 0 | *D* = 0), *k* = 1,2,…, *L*. If test *k* is not 100% specific, it will falsely indicate the presence of disease in individuals known to be healthy. The proportion of positive results among healthy individuals is known as the false-positive rate (FPR_*k*_ = 1 − Sp_*k*_).

### 2.2. Stratification

According to Singer et al. [27], considering the assumption of the model of Hui and Walter [24] in which the population is divided into *V* strata with different disease prevalences (*ξ*_*v*_ = *P*_*v*_(*D* = 1); *v* = 1,2,…, *V*), but with similar performance of the tests among strata (Se_*k*_, Sp_*k*_; *k* = 1,2,…, *L*), the likelihood function of the observed data can be described as

(1) L(θ)=∏v=1V∏i=1nv[ξv∏k=1LSektikv(1−Sek)(1−tikv)  +(1−ξv)∏k=1LSpk(1−tikv)(1−Spk)tikv],

where, ***θ*** = (*ξ*_*v*_, Se_*k*_, Sp_*k*_), *n*_*v*_ is the number of known subjects in the *v*th stratum, and *t*_*ikv*_ is the observed result of the *k*th test in the *v*th stratum to *i*th individual.

When none of these *L* diagnostic tests can be considered to be the gold standard for the disease in question, the true but unknown health status of the *i*th subject (0: healthy or 1: sick) in the *v*th stratum, called *Y*_*v*_, can be modeled from a Bernoulli(*τ*_*iv*_) distribution with success probability *τ*_*iv*_ = *P*_*v*_(*D* = 1 | *T*_*i*1_ = *t*_*i*1_,…, *T*_*iL*_ = *t*_*iL*_). Then some algebraic work is given by

(2) τiv=(ξv∏k=1LSektikv(1−Sek)(1−tikv))/   (ξv∏k=1LSektikv(1−Sek)(1−tikv)   +(1−ξv)∏k=1LSpk(1−tikv)(1−Spk)tikv).

Combining the likelihood function of observed data (1) with the likelihood function of latent variable *Y* = (*Y*_1_, *Y*_2_,…, *Y*_*V*_), after some algebra, we obtain the augmented likelihood function of the latent class model for the general case of *L* conditionally independent tests and *V* strata, given by

(3) 𝕃(θ)=∏v=1V∏i=1nv∏k=1L[ξvSektikv(1−Sek)(1−tikv)]yiv×[(1−ξv)Spk(1−tikv)(1−Spk)tikv](1−yiv).

### 2.3. Including Covariates

 The model structure including *M* covariates can be constructed by linking the covariate matrix **W** = (**W**_1_, **W**_2_,…, **W**_*M*_) to the mean function of the response variable *g*(***θ*** | *𝔇*) from the linear predictor ***ν*** = **W**′**η** according to the relation ***θ*** = *g*(***ν***) = *g* ^−1^(**W**′**η**), where *g* is a monotone and differentiable function, *g* ^−1^ is a link function, ***θ*** = (*ξ*_*v*_, Se_*k*_, Sp_*k*_) is the vector of original parameters, and **η** is the new vector of parameters of dimension *p*.

On the basis of the logit link function, the relations of the vector of covariates *W*_*c*_  (*c* = 1,2,…, *M*) with the original parameters of interest *θ* are given by

(4) $$\begin{array}{c} \xi_{v}=g^{-1}\left(\nu_{vc}\right)=\frac{\exp\left\{\alpha_{v}+\sum_{c=1}^{M}\gamma_{vc}W_{ic}\right\}}{\left(1+\exp\left\{\alpha_{v}+\sum_{c=1}^{M}\gamma_{vc}W_{ic}\right\}\right)};\\ \text{S}{\text{e}}_{k}=g^{-1}\left(\nu_{1kc}\right)=\frac{\exp\left\{\alpha_{1k}+\sum_{c=1}^{M}\gamma_{1kc}W_{ic}\right\}}{\left(1+\exp\left\{\alpha_{1k}+\sum_{c=1}^{M}\gamma_{1kc}W_{ic}\right\}\right)};\\ \text{S}{\text{p}}_{k}=g^{-1}\left(\nu_{0kc}\right)=\frac{\exp\left\{\alpha_{0k}+\sum_{c=1}^{M}\gamma_{0kc}W_{ic}\right\}}{\left(1+\exp\left\{\alpha_{0k}+\sum_{c=1}^{M}\gamma_{0kc}W_{ic}\right\}\right)}, \end{array}$$

where, *α*_*v*_, *α*_1*k*_ and *α*_0*k*_, correspond to the intercepts of the logit link functions (4) for the prevalence in the *v*th stratum and sensitivity and specificity of the *k*th test, respectively (*v* = 1,2,…, *V*, *k* = 1,2,…, *L*), that is, the estimates of these parameters when all covariates are null. We have *γ*_1*kc*_ and *γ*_0*kc*_ indicating how much the *c*th covariate influences the sensitivity and specificity, of the *k*th test, respectively and *γ*_*vc*_ the disease prevalence in the *v*th stratum (*c* = 1,2,…, *M*). Therefore, the new vector of parameters **η** = (*α*_*v*_, *α*_1*k*_, *α*_0*k*_, *γ*_*vc*_, *γ*_1*kc*_, *γ*_0*kc*_) will have dimension *p* = (*M* + 1)(2*L* + *V*).

Replacement of the logit links (4) with their respective original parameters ***θ*** = (*ξ*_*v*_, Se_*k*_, Sp_*k*_) in the augmented likelihood function (3) results in the augmented likelihood function:

(5) 𝕃(η)∝∏v=1V∏i=1nv[exp⁡{αv+∑c=1MγvcWivc}1+exp⁡{αv+∑c=1MγvcWivc}×∏k=1L(exp⁡{α1k+∑c=1Mγ1kcWivc}1+exp⁡{α1k+∑c=1Mγ1kcWivc})tikv×(1−exp⁡{α1k+∑c=1Mγ1kcWivc}1+exp⁡{α1k+∑c=1Mγ1kcWivc})(1−tikv)]yiv[(1−exp⁡{αv+∑c=1MγvcWivc}1+exp⁡{αv+∑c=1MγvcWivc})  ×∏k=1L(exp⁡{α0k+∑c=1Mγ0kcWivc}1+exp⁡{α0k+∑c=1Mγ0kcWivc})(1−tikv)×(1−exp⁡{α0k+∑c=1Mγ0kcWivc}1+exp⁡{α0k+∑c=1Mγ0kcWivc})tikv](1−yiv).

Model (5) covers a wide spectrum of latent class model. The frequentist framework proposed by Hui and Walter [24], Bayesian approach proposed by Joseph et al. [4] and Martinez et al. [8] are some particular cases of our model (5), when (*L* = 2, *M* = 0, *V* = 2), (*L* = 2, *M* = 0, *V* = 1) and (*L*, *M* = 2, *V* = 1), respectively.

### 2.4. Inference

 For inference, we adopt a fully Bayesian approach. This choice was based on the complex structure of this model, which may have many parameters to be estimated depending on the configuration adopted. Also, if necessary or appropriate, expert opinion may be included in modeling via a prior distribution.

This approach is by no means the only modeling method, but it is the first and natural step that has the advantage of simplifying calculations, especially in view of the computational implementation of the Markov Chain Monte Carlo (MCMC) algorithms.

#### 2.4.1. Priors

 Assuming independence between the parameters of the vector **η** and following Dendukuri and Joseph [35] and Menten et al. [36], we consider the normal distribution *N*(*μ*, *σ* ^2^) to model a prior knowledge about each of the parameters of interest the vector **η**. Since *μ* and *σ* ^2^ hyperparameters known location and scale of the normal distribution:

(6) π(η)=∏v=1V∏k=1L∏c=1MN(μαv,σ2αv)N(μγcv,σ2γcv)    N(μα1k,σ2α1k)N(μγ1kc,σ2γ1kc)    N(μα0k,σ2α0k)N(μγ0kc,σ2γ0kc).

#### 2.4.2. Conditional Posteriors

Irrespective of the form of the priors considered, the joint posterior distributions of the parameters are analytically intractable. We overcome this computational difficulty by using the Metropolis-Hastings algorithm [11], which allows us to simulate observations from nontrivial joint distributions by generating random samples successively from the full conditional distributions for the unknown parameters. The full conditional posterior densities for the parameters which are used in each step of the iterative sampling-based algorithms are given by

(7) $$\begin{array}{c} \alpha_{v}\;|\;\eta_{\left(\alpha_{v}\right)},𝔇\;\sim \;N\left(\mu_{\alpha_{v}},{\sigma^{2}}_{\alpha_{v}}\right)\times \psi \left(\alpha_{v}\right)\\ \gamma_{cv}\;|\;\eta_{\left(\gamma_{cv}\right)},𝔇\;\sim \;N\left(\mu_{\gamma_{cv}},{\sigma^{2}}_{\gamma_{cv}}\right)\times \psi \left(\gamma_{cv}\right)\\ \alpha_{1k}\;|\;\eta_{\left(\alpha_{1k}\right)},𝔇\;\sim \;N\left(\mu_{\alpha_{1k}},{\sigma^{2}}_{\alpha_{1k}}\right)\times \psi \left(\alpha_{1k}\right)\\ \gamma_{1kc}\;|\;\eta_{\left(\gamma_{1kc}\right)},𝔇\;\sim \;N\left(\mu_{\gamma_{1kc}},{\sigma^{2}}_{\gamma_{1kc}}\right)\times \psi \left(\gamma_{1kc}\right)\\ \alpha_{0k}\;|\;\eta_{\left(\alpha_{0k}\right)},𝔇\;\sim \;N\left(\mu_{\alpha_{0k}},{\sigma^{2}}_{\alpha_{0k}}\right)\times \psi \left(\alpha_{0k}\right)\\ \gamma_{0kc}\;|\;\eta_{\left(\gamma_{0kc}\right)},𝔇\;\sim \;N\left(\mu_{\gamma_{0kc}},{\sigma^{2}}_{\gamma_{0kc}}\right)\times \psi \left(\gamma_{0kc}\right), \end{array}$$

where, *ψ*(*α*_*v*_), *ψ*(*γ*_*cv*_), *ψ*(*α*_1*kv*_), *ψ*(*γ*_1*kvc*_), *ψ*(*α*_0*kv*_) and *ψ*(*γ*_0*kvc*_) are given by

(8) ψ(αv)=ψ(γcv)=∏i=1nv[exp⁡{αv+∑c=1MγvcWivc}]yiv ×[1+exp⁡{αv+∑c=1MγvcWivc}](−1)ψ(α1k)=ψ(γ1kc)=∏i=1n[(exp⁡{α1k+∑c=1Mγ1kcWic})tik   ×(1+exp⁡{α1k+∑c=1Mγ1kcWic})(−1)]yiψ(α0k)=ψ(γ0kc)=∏i=1n[(exp⁡{α0k+∑c=1Mγ0kcWic})(1−tik)  ×(1+exp⁡{α0k+∑c=1Mγ0kcWic})(−1)](1−yi).

Convergence of the Metropolis-Hastings algorithm to a stationary distribution was evaluated using the reduction factor proposed by Gelman and Rubin [37].

## 3. Simulation Study

A small simulation study was performed in order to confront the performance of the general model proposed with previous models which can be seem as its the particular cases.

We considered a situation with three tests under investigation (*L* = 3), stratified in two stratums (*V* = 2), in presence of a dichotomous covariate (*M* = 1) for a sample with 150 observations. From (9) and (10) we calculated the probabilities *P*_*v*∣*W*,*D*_ as well as their respective quantity of elements *X*_*v*∣*W*,*D*_, for each combination of results of the *L* tests under investigation in the stratum *v* conditioned on the dichotomous covariate *W* = {0,1} health condition of the subject *D* = {0,1}, which are given by

(9) Pv ∣ W=w,D=1(T1v=t1v,…,TLv=tLv ∣ W=w,D=1)  =∏k=1LSek ∣ wtikv(1−Sek ∣ w)(1−tikv),Pv ∣ W=w,D=0(T1v=t1v,…,TLv=tLv ∣ W=w,D=0)  =∏k=1LSpk ∣ w(1−tikv)(1−Spk ∣ w)tikv,

where Se_*k*∣*w*_ and Sp_*k*∣*w*_ are the sensitivity and specificity rates of the *k*th test on the *w*th covariate level, respectively. And *t*_*ikv*_ is the result of the *k*th test in the *v*th stratum for the *i*th subject:

(10) 𝔼(Xv ∣ W,D)=nv[ξvPv ∣ W=w,D=1(T1v=t1v,…,TLv=tLv ∣ W=w,D=1)+(1−ξv)Pv ∣ W=w,D=0(T1v=t1v,…,TLv=tLv ∣ W=w,D=0)].

For generation of the dataset, we considered prevalence sensitivity and specificity rates as shown in (Table 1). 

The proposed model (5) was adjusted to simulated dataset for four special cases: SSensI (*L* = 3, *V* = 1, *M* = 0); SSensII (*L* = 3, *V* = 2, *M* = 0), SSensIII (*L* = 3, *V* = 1, *M* = 1), and SSensIV (*L* = 3, *V* = 2, *M* = 1).

Following Martinez et al. [8], the hyperparameters of the normal distributions in (6), referring to the respective intercepts, were determined from the respective estimates of ***θ*** = (*ξ*_*v*_, Se_*k*_, Sp_*k*_), obtained from the particular model without covariates considering noninformative Beta priors (Uniform: Beta(1,1)) for each parameter of the vector ***θ***. For instance, in this fitting, the sensitivity of test presented a 95% credible interval of (91.71%–99.99%); thus, the value of the location hyperparameter *μ*_*α*__01_ for the intercept of this test, in the logit model (4), was considered to be the mean of the two results between *e* ^*μ*_*α*_01__^/(1 + *e* ^*μ*_*α*_01__^) = 0.9153 and *e* ^*μ*_*α*_01__^/(1 + *e* ^*μ*_*α*_01__^) = 0.9999, that is, *μ*_*α*_01__ = (2.4 + 9.2)/2 = 5.8. All scale hyperparameters for the intercepts were defined as 50% of the respective location hyperparameter, with *σ*_*α*_01__ = 0.5 × 5.8 = 2.9 for this test. The location and scale hyperparameters, referring to the respective parameters *γ*_1*kc*_, *γ*_0*kc*_, and *γ*_*vc*_, were fixed at 0 and 100.

We considered two parallel MCMC chains of 50,000 iterations were run, with the first 5,000 iterations of each chain being discarded and the remaining iterations being selected at intervals of 45. Thus, a final independent and identically distributed stationary sample with a size equals to 2,000 of conditional posterior distributions (7) was obtained for each particular case (SSensI, SSensII, SSensIII, and SSensIV).

In order to decide for the best model to be fitted, we considered the information criteria proposed by Akaike [38] (Akaike's information criteria, AIC) and Schwarz [39] (Bayesian information criterion, BIC). These criteria have been discussed by Raftery [40], Kuha [41], and Posada and Buckley [42], among others. We also consider the deviance information criterion (DIC), which was proposed by Spiegelhalter et al. [43] and subsequently discussed by Kateri et al. [44] and Shriner and Yi [45], among others. Iliopoulos et al. suggested a Bayesian approximation for AIC and BIC [46]. Basically, these criteria quantify the deviation of the fitted model from the observed data. Model presenting the lowest AIC, BIC, and DIC values leads to the best fitting.

The Metropolis-Hastings algorithms, their convergence evaluation, and the AIC, BIC, and DIC criteria can be easily implemented in software **R**, which is freely available at http://www.r-project.org/, by researchers with moderate computer programming knowledge. The codes build for the present study can be requested by e-mail from the authors.

In addition to presenting estimates closer to the nominal (Table 1), the general model proposed demonstrated, from the information criteria, superior performance than the structures without stratification of the population and the absence of covariates (see Table 2).

## 4. Application: Chagas Disease Data

To illustrate the proposed model, we consider a study on the performance of diagnostic tests used in screening for Chagas disease. A sample of 90 blood donors from a blood center in the region of Triângulo Mineiro, Brazil, was considered.

The participants were selected randomly from the three serology strata (SI: 30 donors selected among those with positive serology; SII: 30 donors selected among those with negative serology; SIII: 30 donors selected among those with inconclusive serology in the ELISA screening test). This study was approved by the UFTM Ethics Committee (protocol number. 464).

The ELISA test used for Chagas' disease screening at the time of blood donation was repeated and the participants were submitted to the following five other diagnostic tests: four serological tests including indirect immunofluorescence (IIF), indirect hemagglutination (IHA), which uses red blood cells covered with soluble *T. cruzi* antigen (performed at the Central Laboratory of UFTM), conventional and recombinant ELISA, which use CRA—and FRA—specific membrane antigens of *T. cruzi* (kit from Biomanguinhos Laboratory Fundação Oswaldo Cruz/FIOCRUZ, Brazilian Ministry of Health). The fifth test was the Hemoculture a parasitological test (blood culture in *T. cruzi*-specific) which is 100% specific. However, none of these tests is a gold standard for the detection of Chagas' disease.

 In addition to the application of these six tests, the following epidemiological data were obtained from the donor records in order to evaluate their influence on the performance parameters of the diagnostic tests: age (≤30, >30 years), gender (male, female), and presence (yes, no) of at least two of three epidemiological risk factors (origin from an endemic region, a contact with the vector transmitting Chagas' disease, and a family history of Chagas' disease).

On the basis of a practical need, where it is desired to estimate the prevalence rate of *T. cruzi* infection among donors serologically inconclusive, we consider the serology at the time of donation as a stratifying factor in our model, where the first stratum (SI) is composed of donors with negative or positive serology and the second stratum (SII) is composed of donors with inconclusive serology at the time of screening.

However, to judge whether this decision was adequate, we consider here three particular cases of the model (5), hereafter called model ModI (*V* = 1: no stratification), ModII (*V* = 2: two strata, SI: negative or positive serology at the time of screening; SII: inconclusive serology), and ModIII (*V* = 3: three strata, SI: negative serology at the time of screening; SII: positive serology; SIII: inconclusive serology), in all cases, we consider six tests (*L* = 6) and three covariates (*M* = 3).

The location and scale hyperparameters for model ModII were obtained as described in Section 3 except for the *α*_04_ related to blood culture test (HEMO) which has a specificity of 100% And they are shown in Table 3.

### 4.1. Model Summary

 Following the sensitivity study, based on the Bayesian approximation of AIC and BIC criteria as well as on the DIC, the model with stratification of the population structure showed superior performance to the structure without stratification as we can see from Table 4.

The results reported and discussed here refer to the posterior inferences obtained for the particular case ModII (*L* = 6,  *M* = 3,  *V* = 2) of the proposed model (5), which better fits the data according our model comparison criteria, and then it is considered as our working model. 

Table 5 shows the estimates of the 56 parameters regarding the effects of the *c*th covariate: age, gender, and the presence of at least two of three epidemiological risk factors (origin from an endemic region, a contact with the vector transmitting Chagas' disease, and a family history of Chagas' disease) on the sensitivity (*γ*_1*kc*_) and specificity (*γ*_0*kc*_) of the *k*th diagnostic test and on disease prevalence (*γ*_*vc*_) in the *v*th stratum. The posterior probability estimates most distant from zero indicate an effect of the *c*th covariate, with these estimates being significant when the 95% credible interval (*P*2.5%; *P*97.5%) obtained for each parameter does not contain zero.

With respect to the effect of the *c*th covariate on the original parameters *θ* = (*ξ*_*v*_, Se_*k*_, Sp_*k*_), we observed a significant effect of all covariates on the specificity (*β*_*ck*_) of all tests, except for blood culture, which is 100% specific according to expert opinion. Regarding the prevalence of Chagas' disease, a significant effect of all covariates *γ*_*cv*_ on all strata was observed, except for the age and presence of at least two of three risk factors epidemiological (origin from an endemic region, contact with the vector transmitting the disease, and family history of Chagas' disease) covariates in the stratum II (inconclusive serology, see, Table 5).

The following covariates exerted nonsignificant effects on the sensitivity of the tests: presence of at least two of three risk factors epidemiological (yes, no) for ELISA, blood culture (HEMO), and rec-ELISA; age group (≤ 30;> 30 years) for blood culture and rec-ELISA. On the other hand, the gender (male, female) had a significant effect on the sensitivity of all tests (see Table 5).

### 4.2. Practical Results

Table 6 shows the estimates of the original parameters, sensitivity (Se_*k*_), and specificity (Sp_*k*_) for the *k*th test.

The estimates of disease prevalence (*ξ*_*v*_) in the *v*th stratum are shown in Table 7. In both tables, the results are presented according to each level of the three analyzed covariates. In summary, the lowest sensitivity was observed for the blood culture test (HEMO) in donors younger than 30 years (29.59%). And the highest sensitivity was observed for the ELISA in female donors older than 30 years (99.53%). Despite closely similar nominal values, the sensitivity rates of the serological tests (IIF, IHA, and c-ELISA) were significantly higher in donors older than 30 years, female donors, and donors with presence of at least two of three risk factors epidemiological. In contrast, the blood culture test (HEMO) and the rec-ELISA, presented higher sensitivity only for female donors. The ELISA, IIF, IHA, c-ELISA, and rec-ELISA tests were found to be significantly more specific in donors younger than 30 years, male donors, and donors with presence of at least two of three risk factors epidemiological.

Overall, in the present study, sensitivity rates of 99.25%, 99.12% and 99.13%, and specificity of 95.89%, 99.30% and 99.36% were obtained for ELISA, IHA, and IIF tests, respectively. However, analysis of the estimates obtained with the present model according to each covariate level resulted in sensitivity rates of 98.77%, 98.52%, and 98.56%, respectively, for donors younger than 30 years, of 98.76%, 98.58%, and 98.60% for male donors, and of 98.78%, 98.55%, and 98.56% for donors with presence of at least two of three risk factors epidemiological. Specificity rates were 79.52%, 95.88%, and 96.05%, respectively, for donors older than 30 years; 76.04%, 95.70%, and 95.592% for female donors, and 75.92%, 95.60%, and 95.61% for donors without the presence of at least two of three risk factors epidemiological (see Table 6).

With respect to the prevalence of Chagas' disease, an overall estimate of 12.77% was observed among donors with inconclusive serology in the screening test at the time of blood donation (stratum II). Considering the levels of covariates, we observed that the prevalence of Chagas disease was significantly higher in donors older than 30 years, female and the presence of at least two of three risk factors epidemiological (see Table 7).

Particularly, the estimated rate of chagasic infection of 12.77% among donors with inconclusive results in the serological screening test (stratum II) indicates that 87.23% of these donors do not have Chagas' disease. This result is below the rates reported by Furuchó et al. [47] who observed that 20.5% of donors in the inconclusive group were positive for Chagas' disease, that is, 79.5% of donors with inconclusive serology in the screening test do not have the disease.

 The mean false-positive rate (1 − Sp), which estimates the probability of the test being positive given that the individual is healthy and excluding the blood culture test, with a false-positive rate of 0%, was lower for rec-ELISA testing (0.58%) and higher for c-ELISA (12.84%), whereas the mean false-negative rate (1 − Se), which estimates the probability of a test being negative given the individual has the disease, was lower for c-ELISA (0.84%) and higher for blood culture (55.23%). Analysis of the parallel testing scheme, indicated for urgent cases or for quality control as done at blood banks, in which the set of tests is considered to be positive when at least one of *L* tests presents a positive result, showed lower false-positive rates of 13.35% and 5.79% for the sets of serological tests performed using the tests conducted at the Central Laboratory of UFTM (Sit2:ELISA, IIF, IHA) and the Biomanguinhos-FIOCRUZ kit (Sit4:c-ELISA and rec-ELISA), with false-negative rates of 0.0075% and 0.0002%, respectively. For the serial testing scheme in which the set of tests is considered to be positive when all tests performed are positive, the false-positive rates were 0.073% and 0.0002% for the same serological test sets (Sit2 and Sit4), respectively.

Overall analysis showed that the probability of predicting that an individual is truly infected, given each combination of the results of the six tests, is 100% for each of the two strata always when the blood culture result is positive, irrespective of the results of the other tests since blood culture is 100% specific. When three tests are positive and three tests are negative, excluding the positive blood culture result, the mean probability of predicting that an individual is truly infected is 99.94% for stratum II.

## 5. Final Comments

 To the best of our knowledge, this is the first study proposing a general Bayesian latent class model as the model (5) based on MCMC algorithms for the evaluation of the performance of *L* conditionally independent tests considering the general case of *M* covariates and *V* strata according to the assumption of the proposed model by Hui and Walter [24]. The true health condition of the subject (sick or healthy) in the *v*th stratum is determined by the latent variable *Y*_*v*_ with a Bernoulli (*τ*_*v*_) distribution instead of a gold standard, which is not available in some case such as for the Chagas' disease.

The general model proposed is very flexible and includes all possible configurations for *L* tests, *M* covariates, and *V* strata as particular cases. Besides, it has as particular cases the models proposed by Hui and Walet [24], Joseph et al. [4], and Martinez et al. [8, 23].

Our simulation study finds, based on the information criteria AIC, BIC and DIC, superior performance of the overall structure proposal in comparison with its particular cases (without stratification and/or without covariates) was achieved.

The ModII presented a better fitting for the Chagas' disease dataset than ModI and ModIII, corroborating the initial idea of considering a two-stratum structure (SI: negative or positive serology; SII: inconclusive serology) instead of three strata (SI: negative; SII: positive and SIII: inconclusive serology) or no stratification (SI: negative or positive or inconclusive serology).

According to Swartz et al., a strong correlation between posterior parameters may impair convergence of the algorithm within a reasonable computation time, a fact not observed for the present modeling fitted to the Chagas' data discussed here [48].

Although the latent class structure is not new in studies on diagnostic evaluation in the absence of a gold standard, in the specific case of screening tests for Chagas' disease among blood donors, there is a lack of studies in the literature regarding more elaborate models to estimate the sensitivity and specificity of diagnostic tests and disease prevalence. In most cases, the proposed models estimate these parameters individually for each test and/or considers an imperfect test as gold standard.

The performance parameters of the tests, such as sensitivity and specificity, reached values close to 100% depending on the level of some covariates, a finding supporting the need for a model structure containing covariates to evaluate the performance of multiple conditionally independent diagnostic tests. Particularly, the technique of stratifying the population according to different disease prevalence rates does not add further marked complexity to the model but makes the model more flexible and comprehensive. It should be emphasized that the probability of predicting that an individual is truly infected or healthy is strongly influenced by disease prevalence rates and may widely differ between strata as observed in the present study. Thus, the lack of stratification in the model may contribute to invalidated estimates of these important predictors to predict the presence or absence of a disease given the results of the combinations of the tests used, such as the diagnosis of Chagas' disease in blood donors.

The probability of predicting that an individual is truly infected provides interesting information that contributes to the regarding the true health status of the subject based on the combination of multiple diagnostic tests under evaluation. In the specific case of the diagnosis of Chagas' disease in blood donors, when the results of the five serological tests are combined, repetition of ELISA used at the time of blood donation plus the four other serological tests (IIF, IHA, c-ELISA, rec-ELISA) resulted in a probability of 100% to identify a truly chagasic subject as long as at least four of these tests present positive results in strata II.

When the assumption of conditional independence is violated, that is, the tests are correlated, possible bias may occur in the estimates of the performance parameters of the diagnostic tests as demonstrated by Thibodeau [49] and Vacek [50]. However, according to Georgiadis et al., the model with a conditional independence structure estimates closely similar to those with a conditional dependency model when the performance parameters of the tests are close to 100%, even in the presence of a moderate-to-strong correlation between pairs of tests [51]. Correlated testing framework should be considered further in future on the basis of the proposed model.

According Forman and Engel et al., a latent class model can present weak identifiability if a small sample size in relation to the number of latent classes is observed, with the number of individuals being insufficient to attribute an element to each class and, consequently, to estimate its probabilities. Although we have not faced identifiability problems in our modeling, the problem of weak or lack of identifiability should be the subject of future research in the context of the proposed model [34, 52].

## Figures and Tables

**Table 1:** Settings used for the simulation of artificial data.

	Covariate (*W*)			
	*W* = 0		*W* = 1	
Stratum	*V *		*V*	
Parameter	1	2	1	2
*ξ*_*v*|*W*_	0.10	0.60	0.30	0.90

Se_1|*W*_	0.50		0.60	
Se_2|*W*_	0.75		0.80	
Se_3|*W*_	0.90		0.95	

Sp_1|*W*_	0.60		0.50	
Sp_2|*W*_	0.80		0.75	
Sp_3|*W*_	0.95		0.90	

*ξ*_*v*|*W*_: prevalence rates in *v*th stratum to covariate *W*; Se_*k*|*W*_ and Sp_*k*|*W*_: sensitivity and specificity of *k*th test in *v*th stratum to covariate *W*.

**Table 2:** Selection of models based on the AIC, BIC, and DIC.

Model	*p*	AIC	BIC	DIC
SSensI: (*L* = 3, *V* = 1, *M* = 0)	7	1108483.0	1108514.0	1125999.0
SSensII: (*L* = 3, *V* = 2, *M* = 0)	8	1069892.0	1069927.0	1125833.0
SSensIII: (*L* = 3, *V* = 1, *M* = 1)	14	308389.8	308451.5	309236.4
SSensIV: (*L* = 3, *V* = 2, *M* = 1)	16	167959.7	168030.1	46198.1

*p*: number of parameters in the model.

**Table 3:** Location and scale hyperparameter values of the normal prior distributions (6) for model ModII (*L* = 6, *M* = 3, *V* = 2).

Sensitivity	*α*_1*k*_ ~ *N*(5.8,2.0)	*k* = 1,2, 3,5, 6
	*α*_14_ ~ *N*(−0.5,0.25)	
	*γ*_1*kc*_ ~ *N*(0,100)	*c* = 1,2, 3, *k* = 1,2, 3,4, 5,6

Specificity	*α*_0*k*_ ~ *N*(3.5,2.0)	*k* = 1
	*α*_0*k*_ ~ *N*(6.5,3)	*k* = 2,3, 6
	*α*_0*k*_ ~ *N*(100,1*E* − 100)	*k* = 4
	*α*_0*k*_ ~ *N*(2,1)	*k* = 5
	*γ*_0*kc*_ ~ *N*(0,100)	*c* = 1,2, 3, *k* = 1,2, 3,5, 6
	*γ*_0*kc*_ ~ *N*(0,0)	*c* = 1,2, 3, *k* = 4

Prevalence	*α*_*v*_ ~ *N*(0.5,0.25)	*v* = 1
	*α*_*v*_ ~ *N*(−2,1)	*v* = 2
	*γ*_*vc*_ ~ *N*(0,100)	*c* = 1,2, 3, *v* = 1,2

*c* = 1: AGE-age (≤30, >30 years); *c* = 2: GEN-gender (male, female); *c* = 3: RFE-presence of at least two of three risk factors epidemiological (origin from an endemic region, contact with the vector transmitting the disease and family history of Chagas' disease); Stratum I (*v* = 1) donors with negative or positive serology and Stratum II (*v* = 2) with inconclusive serology in the screening test; *γ*_1*kc*_: effect of the *c*th covariate on the sensitivity of *k*th test; *γ*_0*kc*_: effect of the *c*th covariate on the specificity of *k*th test; *γ*_*vc*_: effect of the *c*th covariate on disease prevalence in *v*th stratum.

**Table 4:** Selection of models based on the AIC, BIC, and DIC.

Model	*p*	AIC	BIC	DIC
ModI: (*L* = 6, *M* = 3, *V* = 1)	52	813.09	943.08	719.88
ModII: (*L* = 6, *M* = 3, *V* = 2)	56	511.39	651.38	402.03
ModIII: (*L* = 6, *M* = 3, *V* = 3)	60	756.49	906.48	638.13

*p*: number of parameters in the model.

**Table 5:** Summary of the posterior probability estimates obtained for the parameters that measure the effect of the *c*th covariate on the sensitivity and specificity of *k*th test and on disease prevalence in *v*th stratum, using model ModII (*L* = 6, *M* = 3, *V* = 2).

Sensitivity (*α*_*ck*_)					Specificity (*β*_*ck*_)				
Test	Parameter	Mean	*P*2.5%	*P*97.5%	Test	Parameter	Mean	*P*2.5%	*P*97.5%
ELISA	*α*_11_	5.91	3.03	8.79	ELISA	*α*_01_	3.47	1.80	5.10
	*γ*_111_	0.50	0.02	0.96		*γ*_011_	−1.92	−2.79	−0.94
	*γ*_112_	0.51	0.03	1.00		*γ*_012_	−2.10	−2.90	−1.14
	*γ*_113_	0.50	−0.01	0.96		*γ*_013_	−2.12	−2.89	−1.11
IIF	*α*_12_	5.79	2.89	8.82	IIF	*α*_02_	6.51	3.01	9.73
	*γ*_121_	0.51	0.04	0.99		*γ*_021_	−2.08	−2.89	−1.02
	*γ*_122_	0.49	0.00	0.98		*γ*_022_	−2.12	−2.89	−1.16
	*γ*_123_	0.50	0.02	1.00		*γ*_023_	−2.12	−2.89	−1.10

IHA	*α*_13_	5.77	2.76	8.65	IHA	*α*_03_	6.56	2.88	10.01
	*γ*_131_	0.51	0.03	0.99		*γ*_031_	−2.10	−2.86	−1.05
	*γ*_132_	0.49	0.01	0.97		*γ*_032_	−2.11	−2.91	−1.11
	*γ*_133_	0.51	0.04	0.98		*γ*_033_	−2.12	−2.85	−1.15

HEMO	*α*_14_	−0.50	−0.78	−0.22	HEMO	*α*_04_	100	100	100
	*γ*_141_	0.38	−0.06	0.84		*γ*_041_	0	0	0
	*γ*_142_	0.45	0.02	0.89		*γ*_042_	0	0	0
	*γ*_143_	0.31	−0.12	0.74		*γ*_043_	0	0	0

c-ELISA	*α*_15_	5.76	2.87	8.75	c-ELISA	*α*_05_	2.00	1.01	2.97
	*γ*_151_	0.51	0.02	1.00		*γ*_051_	−1.91	−2.77	−0.94
	*γ*_152_	0.49	0.04	0.97		*γ*_052_	−2.11	−2.90	−1.07
	*γ*_153_	0.49	0.00	0.98		*γ*_053_	−2.08	−2.85	−1.11

rec-ELISA	*α*_16_	5.72	2.82	8.70	rec-ELISA	*α*_06_	6.50	3.16	10.03
	*γ*_161_	0.21	−0.18	0.74		*γ*_061_	−2.08	−2.87	−1.05
	*γ*_162_	0.54	0.07	1.00		*γ*_062_	−2.09	−2.90	−1.03
	*γ*_163_	0.46	−0.05	0.97		*γ*_063_	−2.11	−2.88	−1.09

Prevalence (*γ*_*cv*_)									
Stratum	Parameter	Mean	*P*2.5%	*P*97.5%	Stratum	Parameter	Mean	*P*2.5%	*P*97.5%

I	*α*_1_	0.50	0.21	0.81	II	*α*_2_	−2.01	−2.92	−1.03
	*γ*_11_	0.51	0.11	0.91		*γ*_21_	0.41	−0.04	0.84
	*γ*_12_	0.43	0.00	0.85		*γ*_22_	0.49	0.03	0.94
	*γ*_13_	0.69	0.28	1.12		*γ*_23_	0.43	−0.02	0.89

*P*2.5%: 2.5th percentile; *P*97.5%: 97.5th percentile; ELISA: enzyme-linked immunosorbent assay; IIF: indirect immunofluorescence; IHA: indirect hemagglutination; HEMO: blood culture; c-ELISA: conventional ELISA using the Biomanguinhos kit; rec-ELISA (recombinant ELISA using the Biomanguinhos kit and CRA and FRA antigens); *c* = 1: AGE-age (≤30, >30 years); *c* = 2: GEN-gender (male, female); *c* = 3: RFE-presence of at least two of three risk factors epidemiological (origin from an endemic region, contact with the vector transmitting the disease, and family history of Chagas' disease); Stratum I (*v* = 1) donors with negative or positive serology and Stratum II (*v* = 2) with inconclusive serology in the screening test; *γ*_1*kc*_: effect of the *c*th covariate on the sensitivity of *k*th test; *γ*_0*kc*_: effect of the *c*th covariate on the specificity of *k*th test; *γ*_*vc*_: effect of the *c*th covariate on disease prevalence in *v*th stratum.

**Table 6:** Sensitivity and specificity of the six diagnostic tests according to each level of the three covariates obtained with model ModII (*L* = 6, *M* = 3, *V* = 2).

		Sensitivity			Specificity		
Test	Covariates	Mean	*P*2.5%	*P*97.5%	Mean	*P*2.5%	*P*97.5%
ELISA	General	0.9925	0.9537	0.9998	0.9589	0.8577	0.9940
	AGE- ≤ 30 years	0.9877	0.9261	0.9998	0.9919	0.9614	0.9993
	AGE- > 30 years	0.9953	0.9704	0.9999	0.7952	0.5007	0.9647
	GEN-male	0.9876	0.9255	0.9998	0.9941	0.9771	0.9994
	GEN-female	0.9953	0.9707	0.9999	0.7604	0.3585	0.9609
	RFE-no	0.9878	0.9252	0.9998	0.9941	0.9740	0.9994
	RFE-yes	0.9952	0.9713	0.9999	0.7592	0.3625	0.9566

IIF	General	0.9913	0.9472	0.9999	0.9936	0.9529	0.9999
	AGE- ≤ 30 years	0.9856	0.9112	0.9998	0.9987	0.9928	1
	AGE- > 30 years	0.9945	0.9677	0.9999	0.9605	0.7202	0.9996
	GEN-male	0.9860	0.9128	0.9998	0.9991	0.9940	1
	GEN-female	0.9943	0.9650	0.9999	0.9559	0.6674	0.9996
	RFE-no	0.9856	0.9171	0.9998	0.9991	0.9937	1
	RFE-yes	0.9945	0.9650	0.9999	0.9561	0.6845	0.9996

IHA	General	0.9912	0.9406	0.9998	0.9930	0.9468	1
	AGE- ≤ 30 years	0.9852	0.9038	0.9997	0.9988	0.9924	1
	AGE- > 30 years	0.9945	0.9667	0.9999	0.9588	0.7063	0.9997
	GEN-male	0.9858	0.9087	0.9997	0.9990	0.9932	1
	GEN-female	0.9943	0.9638	0.9999	0.9570	0.6813	0.9997
	RFE-no	0.9855	0.8989	0.9997	0.9990	0.9920	1
	RFE-yes	0.9945	0.9619	0.9999	0.9560	0.6992	0.9997

HEMO	General	0.3782	0.3140	0.4442	1	1	1
	AGE- ≤ 30 years	0.2959	0.1936	0.4110	1	1	1
	AGE- > 30 years	0.4715	0.3537	0.6006	1	1	1
	GEN-male	0.4014	0.3328	0.4677	1	1	1
	GEN-female	0.4467	0.3511	0.5396	1	1	1
	RFE-no	0.3116	0.2077	0.4275	1	1	1
	RFE-yes	0.4526	0.3317	0.5823	1	1	1

c-ELISA	General	0.9911	0.9461	0.9998	0.8703	0.7333	0.9513
	AGE- ≤ 30 years	0.9853	0.9055	0.9997	0.9747	0.9179	0.9951
	AGE- > 30 years	0.9944	0.9630	0.9999	0.5181	0.2540	0.8044
	GEN-male	0.9861	0.9189	0.9997	0.9795	0.9360	0.9954
	GEN-female	0.9941	0.9625	0.9999	0.4744	0.1936	0.7790
	RFE-no	0.9858	0.9175	0.9997	0.9791	0.9345	0.9955
	RFE-yes	0.9942	0.9605	0.9999	0.4805	0.1946	0.7862

rec-ELISA	General	0.9908	0.9435	0.9998	0.9941	0.9591	1
	AGE- ≤ 30 years	0.9886	0.9294	0.9998	0.9990	0.9939	1
	AGE- > 30 years	0.9923	0.9558	0.9999	0.9606	0.7286	0.9996
	GEN-male	0.9848	0.9129	0.9997	0.9991	0.9940	1
	GEN-female	0.9942	0.9659	0.9999	0.9588	0.6933	0.9997
	RFE-no	0.9851	0.9073	0.9997	0.9992	0.9944	1
	RFE-yes	0.9941	0.9646	0.9999	0.9590	0.7259	0.9997

*P*2.5%: 2.5th percentile; *P*97.5%: 97.5th percentile; ELISA: enzyme-linked immunosorbent assay; IIF: indirect immunofluorescence; IHA: indirect hemagglutination; HEMO: blood culture; c-ELISA: conventional ELISA using the Biomanguinhos kit; rec-ELISA (recombinant ELISA using the Biomanguinhos kit and CRA and FRA antigens); AGE-age (≤30, >30 years); GEN-gender (male, female); RFE-presence of at least two of three risk factors epidemiological (origin from an endemic region, contact with the vector transmitting the disease, and family history of Chagas' disease).

**Table 7:** Prevalence rates of Chagas' disease in each of the three strata according to each level of the three covariates obtained with model ModII (*L* = 6, *M* = 3, *V* = 2).

Prevalence				
Stratum	Covariates	Mean	*P*2.5%	*P*97.5%
I	General	0.6215	0.5516	0.6915
	AGE ≤ 30 years	0.4975	0.3723	0.6237
	AGE > 30 years	0.7298	0.6277	0.8136
	GEN-male	0.5169	0.3899	0.6380
	GEN-female	0.7138	0.6006	0.8054
	RFE-no	0.4527	0.3238	0.5729
	RFE-yes	0.7634	0.6619	0.8471

II	General	0.1277	0.0512	0.2633
	AGE ≤ 30 years	0.0909	0.0310	0.2022
	AGE > 30 years	0.1811	0.0693	0.3700
	GEN-male	0.0845	0.0276	0.2035
	GEN-female	0.1935	0.0705	0.3837
	RFE-no	0.0895	0.0292	0.1987
	RFE-yes	0.1839	0.0718	0.3770

*P*2.5%: 2.5th percentile; *P*97.5%: 97.5th percentile; *v*th Stratum: I, II (donors with negative or positive serology and inconclusive in the screening test, resp.,); AGE-age (≤30, >30 years); GEN-gender (male, female); RFE-presence of at least two of three risk factors epidemiological (origin from an endemic region, contact with the vector transmitting the disease, and family history of Chagas' disease).
